# Incidence and predictors of pericardial effusion following surgical closure of atrial septal defect in children: A single center experience

**DOI:** 10.3389/fped.2022.882118

**Published:** 2022-08-09

**Authors:** Martina Campisano, Camilla Celani, Alessio Franceschini, Denise Pires Marafon, Silvia Federici, Gianluca Brancaccio, Lorenzo Galletti, Fabrizio De Benedetti, Marcello Chinali, Antonella Insalaco

**Affiliations:** ^1^Division of Pediatric Cardiology, Bambino Gesù Children’s Hospital, Istituti di Ricovero e Cura a Carattere Scientifico (IRCCS), Rome, Italy; ^2^Division of Rheumatology, Bambino Gesù Children’s Hospital, Istituti di Ricovero e Cura a Carattere Scientifico (IRCCS), Rome, Italy

**Keywords:** atrial septal defect, pericardial effusion, body mass index, post-pericardiotomy syndrome, congenital heart disease

## Abstract

**Objectives:**

To evaluate the incidence of pericardial effusion (PE) after surgical atrial septal defect (ASD) closure and to investigate the presence of predictive risk factors for its development.

**Methods:**

We collected data from 203 patients followed at Bambino Gesù Children’s Hospital of Rome who underwent cardiac surgery for ASD repair between January 2015 and September 2019.

**Results:**

A total of 200/203 patients with different types of ASD were included. Patients were divided into two groups: Group 1) 38 (19%) who developed PE and Group 2) 162 (81%) without PE. No differences were noted between the two groups with regard to gender or age at the surgery. Fever in the 48 h after surgery was significantly more frequent in group 1 than in group 2 (23.7 vs. 2.5%; *p* < 0.0001). ECG at discharge showed significant ST-segment elevation in children who developed PE, 24.3 vs. 2.0% in those who did not (*p* < 0.0001). Group 1 patients were divided into two subgroups on the basis of the severity of PE, namely, 31 (81.6%) with mild and 7 (18.4%) with moderate/severe PE. Patients with moderate/severe PE had a significantly higher BMI value (median 19.1 Kg/m^2^) (range 15.9–23.4, *p* = 0.004).

**Conclusion:**

The presence of fever and ST-segment elevation after surgery predicts subsequent development of PE suggesting a closer follow-up for these categories of patients. A higher BMI appears to be associated with a higher risk of moderate/severe PE.

## Introduction

Pericardial effusion (PE) occurs commonly after open heart surgery and may present as a common complication of any cardiac surgery involving an incision of the pericardium with an incidence of 20% of surgically treated patients. PE usually occurs during the first month after operation (1, 2).

PE after cardiac surgery represents the main feature of the post-pericardiotomy syndrome (PPS) that generally includes the following symptoms or signs: fever occurring at least 72 h postoperatively, pericardial or pleural rub, and worsening or recurring anterior chest pain (3).

Due to improvements in the detection of congenital heart disease and surgical techniques, and the consequent increase in the long-term survival, an increasing number of children undergo corrective cardiac surgery. PE after cardiac surgery represents an important cause of morbidity and hospitalization. In most cases, PE has a mild course and does not require invasive treatment. In a small percentage (0.1–8.8%) (4), it leads to cardiac tamponade, requiring pericardiocentesis. Some authors have reported that surgical closure of atrial septal defect (ASD) is associated with a higher risk to develop PE and PPS than other cardiac surgical procedures. The etiology behind this association remains still unclear. It has been previously proposed that the presence of right atrial enlargement and chronic elevation of interatrial pressure seen in ASD defects may play a possible role in the development of PE (5). Among congenital heart disease, ASD has a high frequency with an incidence ranging from 0.3 to 1.64 per 1,000 live birth and is considered as the third commonest type of congenital heart disease (6, 7). Surgical repair occurs mainly during childhood to prevent symptoms of chronic volume overload of the right atrium and ventricle. Corrective surgery for ASD is usually uncomplicated, with PE representing its most significant potential sequela. Nowadays, few studies have analyzed the incidence of PE in a pediatric group with different types of ASD after cardiac surgery. The purpose of this study is to determine the incidence of PE after surgical closure of different types of ASD and to investigate possible predictors of its development.

## Materials and methods

We retrospectively collected 203 patients followed at the Bambino Gesù Children’s Hospital of Rome who underwent cardiac surgery for isolated ASD surgical repair between January 2015 and September 2019. Patients underwent surgical closure due to ineligibility for percutaneous procedures, including not ostium secundum defects, presence of markedly aneurysmal septum or with multiple fenestrations, and scarcity of surrounding tissue.

We collected demographic and clinical variables as reported in Table 1, including gender, age at surgery, body mass index (BMI), presence of comorbidities blood pressure (BP) before surgery, type of ASD, and duration of surgery. Presurgical cardiac parameters were collected through an electrocardiogram (ECG) and complete 2-D Doppler echocardiography.

**TABLE 1:** Demographic and baseline features of patients with pericardial effusion (Group 1) vs. those without pericardial effusion (Group 2).

	All patients	Group 1	Group 2	*P*-value
	*N* = 200	*N* = 38	*N* = 162	
Female^a^	122 (61.0)	21 (55.3)	101 (62.4)	0.42
Age at surgery (years)^b^	4.4 (2.8–7.3)	4.9 (3.3–9.1)	4.3 (2.6–6.7)	0.08
BMI (Kg/m^2^)^b^*	15.2 (14.0–17.1)	15.7 (14.0–18.0)	15.0 (13.9–17.0)	0.61
Weight for age percentiles^b^	33.0 (7.7–71.9)	36.9 (7.8–73.9)	32.1 (7.6–71.2)	0.81
Comorbidity**	67 (33.5)	14 (36.8)	53 (32.7)	0.63
Hypertension^a^	39/173 (22.5)	9/38 (23.6)	30/135 (22.2)	0.83
Echo. pre-surgery^b^				
ASD size (mm)	16.0 (11.0–19.0)	14.0 (9.4–18.5)	16.0 (12.0–19.0)	0.35
EF (%)	69.8 (63.1–74.3)	70.0 (62.6–74.0)	69.4 (63.4–74.8)	0.38
RVFAC (%)	48.0 (43.2–53.8)	48.4 (43.1–54.0)	47.8 (43.2–54.3)	0.56
RVESP (mmHg)	34.6 (24.2–45.1)	34.5 (24.6–46.2)	34.8 (23.2–46.7)	0.92
LVEDa (cm^2^/m^2^)	13.1 (10.3–16.4)	12.9 (10.1–16.3)	13.2 (10.2–16.5)	0.33
ASD type^a^				
OS-ASD	121 (60.5)	21 (55.3)	100 (61.7)	0.46
Sinus-ASD	51 (25.5)	13 (34.2)	38 (23.5)	0.17
OP-ASD	25 (12.5)	4 (10.5)	21 (13.0)	0.79
Un-ASD	3 (1.5)	0 (0.0)	3 (1.8)	1.0
Duration of surgery (min)^b^	47 (34–75)	46 (37–71)	47 (33–75)	0.96

^a^Number (percentage)—Chi-square test or Fisher’s exact test. ^b^Median (1st–3rd quartile)—Mann-Whitney U-test.

BMI, body mass index; Echo., Echocardiogram; ASD, atrial septal defect; EF, ejection fraction; RVFAC, right ventricular fractional area change; RVESP, right ventricular end-systolic pressure estimated from tricuspid regurgitation; LVED-a, left ventricular end-diastolic area index measured from apical 4-chamber view, OS-ASD, ostium secundum ASD; sinus-ASD, sinus venosus ASD; OP-ASD, ostium primum ASD; un-ASD, unroofed coronary sinus ASD.

*As 30 children were below 2 years of age this analysis was performed on 170 children (Group 1 = 35; Group 2 = 135). **Comorbidities include genetic syndromes (14.5%) and other pathological conditions including celiac disease, other cardiac anomalies, esophageal atresia, respiratory disease.

We also collected postsurgical variables, described in Table 2 that reported: the presence of fever in the first 48 h after surgery, laboratory findings ECG within 48 h following surgery, at the time of hospital discharge (3–5 days after surgery), and at patients’ follow-up visit after a median time of 15 days post-surgery. We performed post-surgery echocardiography in all children independently of symptoms (3–5 days after surgery) and, additionally, in children with incident symptoms suggesting PPS. Outpatient follow-up after a median time of 15 days post-surgery.

**TABLE 2:** Post-surgical variables of patients with pericardial effusion (Group 1) vs. those without pericardial effusion (Group 2).

	All patients	Group 1	Group 2	*P*-value
	*N* = 200	*N* = 38	*N* = 162	
Fever^a^	13 (6.5)	9 (23.7)	4 (2.5)	<0.0001
ECG	*n* = 187	*n* = 37	*n* = 150	
ECG HR (bpm)^b^	105 (90–125)	105 (90–128)	104 (94–125)	0.65
RAD^a^	25 (13.4)	3 (8.1)	22 (14.7)	0.42
PR alteration^a^	6 (3.2)	1 (2.7)	5 (3.3)	1.0
ECG Wave-QRS^a^	6 (3.2)	3 (8.1)	3 (2.0)	0.09
ECG ST-T alt^a^	12 (6.4)	9 (24.3)	3 (2.0)	<0.0001
Laboratory findings				
CRP (mg/dl)^b^	3.86 (2.36–5.62)	4.18 (2.71–5.88)	3.76 (2.23–5.53)	0.23
WBC (10^∧3^/μl)^b^	12.2 (9.9–15.1)	12.9 (10.2–15.8)	12.0 (9.8–14.7)	0.30
Neutr (10^∧3^/μl)^b^	9.5 (7.2–12.2)	10.8 (7.6–13.1)	9.3 (7.1–11.9)	0.17

Laboratory parameters of inflammation were obtained in the 24 h following surgery. ^a^Number (percentage)—Chi-square test or Fisher’s exact test. ^b^Median (1st–3rd quartile)—Mann-Whitney U-test.

ECG, electrocardiogram; HR, heart rate; bpm, beats per minute; RAD, right axis deviation; Afib, atrial fibrillation; alt, alterations; CRP, C-reactive protein; WBC, white blood cells; Neutr, neutrophils.

### Echocardiography

An echocardiographic examination was performed with a complete transthoracic echocardiographic study by the Philips echocardiograph Epic cVx model (Philips Medical System, Andover, MA, United States), and images were captured with two-dimensional probes S-8 or X-5, according to the patient’s weight.

The images were digitized, and measurements were taken offline according to the guidelines of the American Society of Echocardiography. We detected conventional parameters from the parasternal long axis window over three consecutive M-mode cycles.

The dimensions of the ASD were obtained mainly from the subcostal Bicaval view, an average of at least 3 measurements was performed.

The presence of PE was analyzed as a categorical variable. The quantification of PE (mild, moderate, and severe) was performed in a semi-quantitative fashion by the mean diastolic dimension measured in diastole in parasternal views, and by analysis of echocardiographic signs of significant hemodynamic impact. The effusion was measured in both long- and short-axis parasternal views. Mild PE was defined as an effusion less than 10 mm and without hemodynamic effect. Moderate PE was defined as a > 10 mm and < 15 mm and no hemodynamic effect; severe PE was defined as an effusion > 15 mm or tamponade physiology (including echocardiographic and clinical signs of tamponade, changes in BP, poor general clinical condition, and breathing difficulty). Echocardiographic signs of significant hemodynamic effect included: right atrial collapse > 1/3 of the cardiac cycle, inferior vena cava (IVC) plethora, right ventricular collapse, left ventricular collapse, Doppler respiratory variation of the transmitral (> 25%) and/or transtricuspid (> 40%) diastolic flow.

### Blood pressure

Blood pressure was measured in outpatient settings before surgery. Each measurement was placed in a pressure class according to the age of the subject and the centile of height. To carry out an adequate assessment, the sample was divided into hypertensive patients if systolic BP and/or diastolic BP were found to be greater than or equal to 95°C for gender, age, and height on at least three separate measurements (8).

### Statistical analysis

Qualitative variables were expressed as absolute frequencies and percentages. Proportions were compared by the Chi-square test or Fisher’s exact test, as appropriate. Quantitative variables, reported as medians and interquartile ranges (IQR: 1st–3rd quartile) or ranges, were analyzed using the Mann-Whitney *U*-test for unmatched groups.

All statistical tests were two-sided; a *p*-value < 0.05 was considered statistically significant. The analyses were performed, and graphs were generated using Stata 15.1 software (StataCorp LLC, College Station, Texas, United States, 2017) and GraphPad Prism 5.0 (GraphPad Software, Inc., San Diego, CA).

The association of fever in the 48 h after surgery and of ST-segment elevation with the risk of developing PE was quantified by calculating the risk ratio and 95°confidence intervals.

## Results

### Study population

We excluded from the analysis three patients who did not have adequate echocardiographic follow-up data. Accordingly, data from 200 patients, 122 (61%) females were acquired. A total of 162 patients (81%) did not present with PE (group 2) and 38 patients (19%) developed PE (group 1). The median time for the development of PE in group 1 was 15 days after the surgical procedure (IQR 6–20). Seven patients developed PPS (18.4%) with a median time of 38 days after surgery.

Table 1 summarizes the demographic, clinical, and surgical characteristics of the whole set of patients and of those in groups 1 and 2. In the 200 patients, the median age at the time of the surgery was 4.4 years (IQR 2.8–7.3). The population had a median BMI of 15.2 kg/m^2^ (IQR 14.0–17.1). As BMI is not considered reliable in children aged < 2 years, we excluded 30 children aged < 2 years. Therefore, we repeated the analysis on the whole group of patients using weight for age percentiles: the median weight for age percentiles was 33.0 (IQR 7.7–71.9). Comorbidities were present in 33.5% and included genetic diseases, such as DiGeorge syndrome, Cystic Fibrosis, Down syndrome, Noonan syndrome, Kabuki syndrome, Sotos syndrome, and other conditions.

**FIGURE 1:**
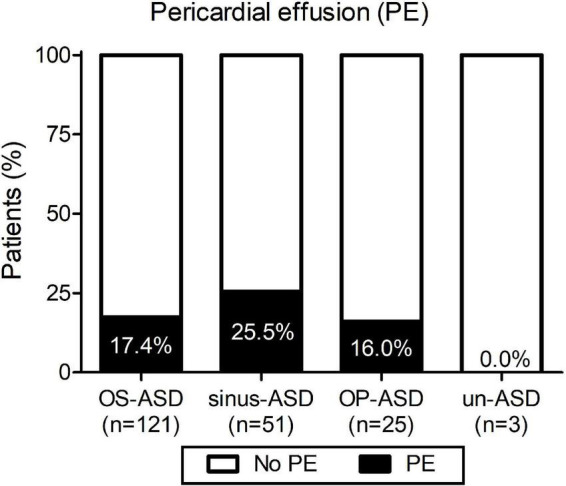
Incidence of pericardial effusion (PE) according to the type of atrial septal defect: ostium secundum atrial septal defect (OS-ASD), sinus venosus ASD (sinus-ASD), ostium primum ASD (OP-ASD), and unroofed coronary sinus ASD (un-ASD).

As shown in Figure 1, different ASD types were reported and the incidence of post-surgical PE was not significantly associated with any of the types of ASD, although a minor trend for a higher probability was observed for sinus-ASD.

### Variables associated with the development of pericardial effusion

**FIGURE 2:**
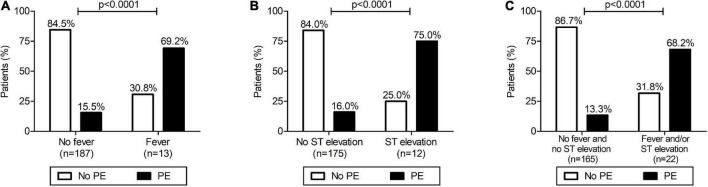
Development of pericardial effusion (PE) after surgical closure of atrial septal defect: relationship with fever **(A)**, ST-segment elevation **(B)** and with fever and/or ST elevation **(C)**. **(A)** Patients with presence of fever in the 48 h after surgery had a significantly higher incidence of PE (Fisher’s exact test: *p* < 0.0001) with a risk ratio for PE of 4.5 (95%CI 2.7–7.3). **(B)** Patients with an ST-segment elevation after surgery had a significantly higher incidence of PE (Fisher’s exact test: *p* < 0.0001) with a risk ratio of 4.7 (95%CI 2.9–7.5). **(C)** Patients with fever in the 48 h after surgery and/or an ST-segment elevation after surgery had a significantly higher incidence of PE (Fisher’s exact test: *p* < 0.0001) with a risk ratio of 5.1 (95%CI 3.2–8.3).

As shown in Tables 1, 2, among the list of demographical, clinical, surgical, and post-surgical variables analyzed, we found a statistically significant difference between group 1 (with PE) and group 2 (without PE) for the presence of fever during the 48 h post-procedure and the presence of ST-segment elevation after cardiac surgery. In detail, patients with fever in the 48 h after surgery had a significantly higher incidence of PE (69.2 vs. 15.5%; *p* < 0.0001) with a risk ratio of 4.5 (95%CI 2.7–7.3) (Figure 2A). ST-segment elevation was present in 9/37 (24.3%) children with PE compared to 3/150 (2.0%) patients without PE, with a risk ratio of 4.7 (95%CI 2.9–7.5; *p* < 0.0001) (Table 2 and Figure 2B). The risk ratio to develop PE for the group of patients with fever after surgery and/or ST-segment elevation compared to those without fever and without ST-segment elevation was 5.1 (95%CI 3.2–8.3; *p* < 0.0001) (Figure 2C).

**FIGURE 3:**
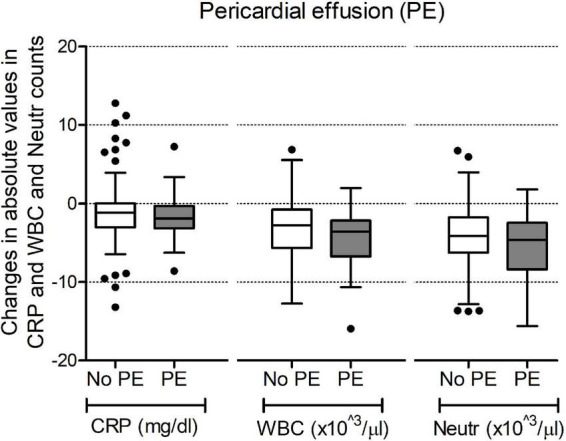
Changes in values of C-reactive protein (CRP), white blood cell counts (WBC) and neutrophil counts (Neutr) during the 72 h after surgery. Data are shown as changes in absolute values in mg/dl for CRP and 10^3^/μl for WBC and Neutr counts.

Markers of inflammation (CRP, WBC, and neutrophil counts), immediately after surgery were similar between groups 1 and 2 (Table 2). As expected, a decrease was noted in the subsequent hours with CRP levels and WBC and neutrophil counts being significantly lower at 72 h compared to the time immediately after surgery [at 72 h median CRP 2.13 mg/dl (IQR 1.18–3.95), median WBC 8.8 10^3^/μL (IQR 7.7–10.7), median neutrophil count 5.2 10^3^/μL (IQR 4.0–6.9)]. We analyzed their changes during the 72 h following surgery and compared the trend over time between group 1 and group 2. As shown in Figure 3, no difference in trends over time was observed.

### Variable associated with the severity of pericardial effusion

Of patients with PE, 31 (81.6%) had mild PE and 7 (18.4%) had moderate/severe PE based on the amount of fluid accumulation and the hemodynamic effect. To verify whether demographical, clinical surgical, and post-surgical variables were associated with the severity of PE, we compared these variables in patients with moderate/severe PE vs. those with mild PE (Table 3).

**TABLE 3:** Features of patients with mild pericardial effusion (PE) vs. patients with moderate/severe pericardial effusion.

	mild PE	moderate/severe PE	*P*-value
	*N* = 31	*N* = 7	
Female^a^	19 (61.3)	2 (28.6)	0.21
Age at surgery (years)^b^	4.5 (0.5–17.0)	6.9 (1.2–17.3)	0.24
BMI (Kg/m^2^)^b^*	14.7 (11.5–20.0) *n* = 29	19.1 (15.9–23.4) *n* = 6	0.004
Weight for age percentiles^b^	24.2 (0.1–95.6)	89.6 (12.5–92.5)	0.014
Comorbidity^a^ Hypertension^a^	10 (32.3) 8 (25.8%)	4 (57.1) 1 (14.2%)	0.39 0.51
Echo. pre-surgery^b^			
ASD size (mm)	16.1 (1.3–25.0)	12.0 (6.5–30.0)	0.44
EF (%)	69.2 (46.4–80.2)	72.1 (62.4–74.6)	0.46
ASD type^a^			
OS-ASD	17 (54.8)	4 (57.1)	1.0
Sinus-ASD	10 (32.3)	3 (42.9)	0.67
OP-ASD	4 (12.9)	0 (0.0)	1.0
Un-ASD	0 (0.0)	0 (0.0)	–
ECC duration (min)^b^	50 (36–75)	45 (38–49)	0.69
Fever post surgery^a^	5 (16.1)	4 (57.1)	0.04
Time to PE (days)^b^	15 (6–20)	15 (6–60)	0.86

^a^Number (percentage)—Chi-square test or Fisher’s exact test. ^b^Median (range)–Mann-Whitney U-test.

BMI, body mass index; Echo., Echocardiogram; ASD, atrial septal defect; EF, ejection fraction; OS-ASD, ostium secundum ASD; sinus-ASD, sinus venosus ASD; OP-ASD, ostium primum ASD; un-ASD, unroofed coronary sinus ASD; ECC, extra-corporeal circulation.

*As 3 children were below 2 years of age this analysis was performed on 29 children with mild PE and 6 children with moderate/severe PE.

**FIGURE 4:**
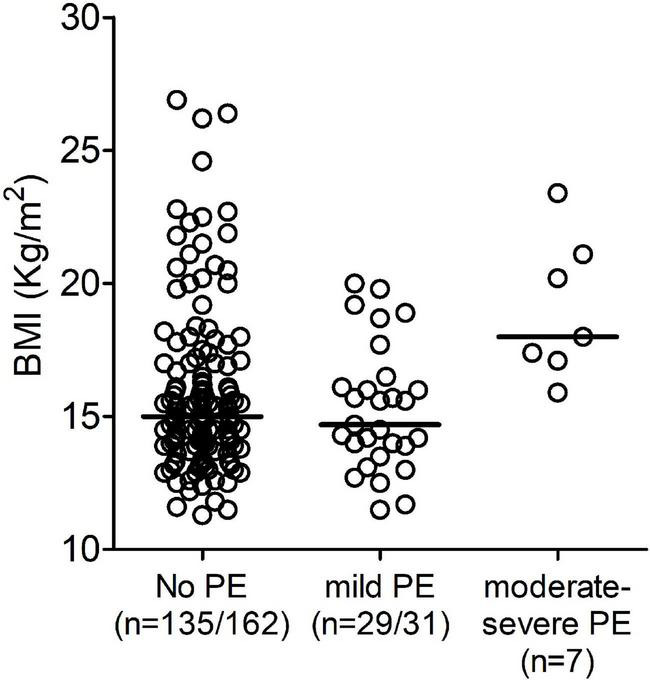
Distribution of the body mass index (BMI) divided into three groups based on the presence and hemodynamic effect of pericardial effusion (PE): No PE, mild PE, and moderate/severe PE.

We found that patients with moderate/severe PE had a significantly higher BMI compared to patients with mild PE. Patients with mild PE had a median BMI of 14.7 kg/m^2^ (range 11.5–20.0), while in the patients with moderate/severe PE BMI was 19.1 kg/m^2^ (range 15.9–23.4; *p* = 0.004) (Table 3 and Figure 4). As mentioned above, BMI is not considered reliable in children aged < 2 years. Therefore, we repeated the analysis using weight for age percentiles. The median was 24.2 in patients with mild PE and 89.6 in patients with moderate/severe PE (*p* = 0.014). The group with moderate/severe PE fever in the 48 h after surgery occurred with a significantly higher incidence compared to the group with mild PE: 57.1% of patients with moderate/severe PE compared to 16.1% of patients with mild PE (*p* = 0.04). There was a trend for a higher age in patients with moderate/severe PE compared to patients with mild PE, but the difference did not reach statistical significance. No significant differences were found in terms of the duration of surgery or median time postoperatively to the diagnosis of PE (median 15 days in the two groups).

Only 3 patients with PE did not receive any treatment. All 35 treated patients received ibuprofen. Colchicine was added in 9/35 patients, most often in patients with moderate/severe PE. Response to treatment was satisfactory in the great majority with only 4 patients requiring pericardiocentesis. There was only one relapse in 1 patient who was subsequently treated with the IL-1 inhibitor anakinra with no further relapses.

## Discussion

Pericardial effusion after pediatric cardiac surgery is a known frequent complication after surgery for any type of congenital heart disease. There has been an important decrease in the prevalence over the last decades with earlier studies reporting a prevalence of PE ranging from 53 to 85% (9). This decrease might be attributed to improved surgical techniques and/or post-surgical care (10).

There are some studies reporting the incidence of PE after cardiac surgery in children. These studies have also attempted to identify possible risk factors associated with the development of PE. However, the data are scanty and in part conflicting and this is possibly related to the nature of the patients enrolled. The majority of pediatric studies reported a prevalence/incidence of PE after cardiac surgery for all types of congenital heart disease, with ASD repair being included among the indications for surgery. In the most recent 4 studies, published in the last 20 years, that overall included more than 2,500 cardiac surgeries, the incidence of PE ranges from 15 to 24% (11–14). All these studies included all types of cardiac surgery. When the relation between the occurrence of PE and the type of cardiac surgery was evaluated, overall, these studies reported that PE occurred more frequently in Fontan type surgery and ASD repair (10, 11). In our study, we chose to focus exclusively on cardiac surgery for ASD repair, with the aim of excluding the confounding effects of the type of cardiac surgery and focusing on clinical/laboratory variables that might help in identifying patients at risk for PE. Incidentally, we have included different types of ASD and did not find evident differences in the incidence of PE according to the ASD type. In the 200 patients enrolled in our study, the incidence of PE was 19%. There are a few studies that report on PE after surgical repair of ASD in children. The incidence in our study is somewhat lower than that reported by Gill et al. in 177 children (27%) or by Heching et al. in 97 children (37%) (3, 15). In a less recent study published in 2001, the incidence of PE is reported as 53% in only 36 children (16). In a study, involving a nationwide analysis of re-admissions for pericarditis following cardiac surgery, the incidence of the event in children was reported as 1.3% over > 33,000 procedures for ASD in 12 years (17). However, as re-admission was the outcome analyzed, this study selected the most severe phenotype.

In our study, the median time postoperatively to the diagnosis of PE is about 15 days (IQR 6–20). The median time of occurrence of PE is similar to that of other studies after all types of cardiac surgery, and in particularly for ASD repair, further supporting the indication of echocardiographic after the surgery even if there are no symptoms, particularly in children. Indeed, we found that 7 patients (3.5%) presented signs and symptoms meeting the diagnostic criteria for a diagnosis of PPS, according to published clinical trials (3, 14). In the two mentioned above studies that reported the incidence of PPS following ASD repair, the results varied considerably from 2.8 to 28% (3, 15). This may reflect the different PPS criteria between studies, as well as the difficulty in making a diagnosis of PPS in children. As an example, children are often too young to complain of chest pain or, even more so, to differentiate it from incisional pain. Therefore, in our study, we decided to focus on PE, which can be identified and quantitated objectively with echocardiography. As mentioned above, we aimed to identify possible predictive factors for the development of PE, using a homogenous group of patients as far as the type of surgery is concerned. Gender, age, duration of surgery, or presence of comorbidities were not predictive of the development of PE. Our data do not confirm the association of the development of PE with female gender and trisomy 21, observed in studies in which all types of surgery were included (11, 12). Older age has been reported as a risk factor for PE in different studies (13, 17, 18). In particular, Engle et al. (18) found in a study on all types of surgery an elevated incidence of PE and PPS in children older than 2 years of age compared with infants younger than 2 years of age (respectively, 27 vs. 3.5%). In our study, the median age was low, 4.4 years, and there was only a trend for older age in children with PE that did not reach statistical significance. When we used the 2 years cut-off, we found an incidence of 10% (3/30) in children < 2 years of age compared to 20.6% (35/170) in the older children, the difference did not achieve statistical significance (*p* = 0.17). When we analyzed post-surgery clinical laboratory and cardiac variables, we found that fever in the 48 h after surgery and ST-segment elevation were more frequent in patients who developed PE. Despite the fact that fever after surgery (until 48 h) is consistent with the inflammatory etiology of this condition, peripheral blood routine chemistry did not show elevated inflammatory markers, such as CRP levels and WBC and neutrophil counts. This apparent discrepancy may be time dependent, i.e., it takes longer for inflammatory markers to rise compared to the appearance of fever. We cannot exclude that measuring markers of inflammation after the latest time point in the study (i.e., 72 h post-surgery) could help in identifying patients at risk for PE.

Furthermore, ST segment alteration was found to be predictive of development of PE with ST-segment elevation after surgery present in 24.3% of patients with PE and in 2.0% of patients without PE, with a relative risk ratio of 4.9. No data are available on the predictive role of fever after surgery and ST-segment elevation. One study that used PPS as the outcome, reported that the presence of a small PE at discharge was associated with the development of PPS (3). In this study ST-segment elevation, and other ECG alterations appeared not to be predictive of the development of PPS. In our study, no association between minor effusion and subsequent PE was observed (data not shown). However, this study used a different outcome PPS with all the above-mentioned potential caveats about identifying PPS in children. The predictive values of these two simple clinical and electrocardiographic parameters on the risk of developing PE would benefit from confirmation in an independent study.

All patients with PE were treated with NSAIDs, and 9 of the patients with moderate/severe PE also received also colchicine. In our center, we do not use preoperative therapy. Some studies have investigated the possibility of preventing PPS and PE with different therapeutic strategies, including aspirin, glucocorticoids, or colchicine (9, 19, 20). Interestingly, none of the approaches proved to be effective in prevention except colchicine. Indeed Brucato et al. in their study demonstrate that colchicine administered during the 72 h following surgery, significantly reduces the incidence of PPS, but did not completely prevent effusions from occurring and some patients have adverse events (21, 22). Given the safety profile in these trials, identification of patients at risk of PE, as we have done in this study, may allow for a better risk/benefit ratio of preventive treatment with colchicine.

We divided the patients with PE into two groups based on the amount of PE and its hemodynamic effect. We found that patients with moderate/severe PE had a higher BMI than those with mild PE.

There are no data in children on the association between PE and high BMI. However, there are some studies that report the correlation between obesity and PE in adults. One suggests that the presence of obesity is associated with the risk of PE after cardiac surgery (23). Another study demonstrates that increasing BMI is a predictor of relapse of PPS (24). A possible explanation for this correlation is that patients with elevated BMI are more vulnerable to developing systemic inflammation and therefore also pericardial inflammation (25). On the contrary, van Osch et al., in their study on 882 adult patients undergoing valve surgery, demonstrated that a higher BMI was associated with a lower risk of PPS (26). The association with high BMI, therefore, remains controversial and may require additional studies.

We acknowledge that our study analyses data from a single center and being a retrospective study we couldn’t exclude other confounding. Another limitation of the study is the time to perform echocardiography. It is possible that PE or PPS could be discovered in another timeframe respect to our, but we think that these cases have poor clinical significance.

## Conclusion

Pericardial effusion remains a common complication after ASD repair. The identification of predictive factors for the development of PE (i.e., fever and/or ST-segment elevation post-surgery) indicates the need for closer follow-up in these patients after discharge, taking also into account that those with severe PE have higher BMI. Our data also suggests the possibility of preventive treatment with colchicine in high-risk categories of patients. This should be established ideally in a controlled trial.

## Data availability statement

The raw data supporting the conclusions of this article will be made available by the authors, without undue reservation.

## Author contributions

MCh, AI, AF, FD, and CC contributed to the conception and design of the study. MCa and DP organized the database. MCh and DP performed the statistical analysis. CC and MCa wrote the first draft of the manuscript. FD, SF, GB, and LG wrote sections of the manuscript. All authors contributed to manuscript revision, read, and approved the submitted version.

## Abbreviations

ASD: atrial septal defect
BMI: body mass index
PPS: post-pericardiotomy syndrome
PE: pericardial effusion
LVM: mass of the left ventricle
LVH: left ventricular hypertrophy.
